# Supplementary material for: Exploiting pleiotropy to enhance variant discovery with functional false discovery rates
Source: Nat Comput Sci. 2025 Aug 22;5(9):769–81. doi: 10.1038/s43588-025-00852-3 (PMC12457191; doi:10.1038/s43588-025-00852-3)
Supplement: Supplementary file 1 — Supplementary Figs. 1–25 and Tables 1–4. [file 43588_2025_852_MOESM1_ESM.pdf]

nature computational science

Supplementary information https://doi.org/10.1038/s43588-025-00852-3

Exploiting pleiotropy to enhance variant discovery with functional false discovery rates

In the format provided by the authors and unedited

Contents

1 Supplementary tables 2

2 Supplementary figures 5

1 Supplementary tables

							Informative traits					
							Rheumatoid Arthritis		Hypothyroidism			
Disease	Chr	rsid	Gene	MAF	β	P	β	P	β	P	P_f	Discovery P
ATH (R12)	1	rs6679677:C>A	*RSBN1*	0.142	0.53	2.75×10^{-15}	0.59	3.10×10^{-149}	0.38	1.44×10^{-154}	1.18×10^{-19}	5.37×10^{-44} [1]
	12	rs7137828:C>T	*ATXN2*	0.410	-0.23	2.04×10^{-5}	-0.08	5.30×10^{-7}	-0.22	1.70×10^{-112}	3.29×10^{-9}	4.72×10^{-25} [1]
	2	rs3087243:G>A	*CTLA4*	0.333	-0.21	2.26×10^{-4}	-0.14	9.20×10^{-20}	-0.18	9.42×10^{-77}	5.71×10^{-9}	3.62×10^{-14} [1]
JIA (R5)	12	rs653178:C>T	*ATXN2*	0.470	0.05	1.83×10^{-5}	-0.08	5.60×10^{-7}	-0.21	1.07×10^{-111}	2.88×10^{-9}	1.82×10^{-9} [2]
	1	rs6679677:C>A	*RSBN1*	0.179	0.07	1.66×10^{-3}	0.59	3.10×10^{-149}	0.38	1.44×10^{-154}	1.81×10^{-8}	9.18×10^{-14} [2]
MYO (R12)	1	rs6679677:C>A	*RSBN1*	0.145	0.28	4.97×10^{-6}	0.59	3.10×10^{-149}	0.38	1.44×10^{-154}	1.03×10^{-8}	2.00×10^{-7} [3]
SLE (R7)	7	rs3778754:C>G	*IRF5*	0.429	0.34	9.09×10^{-13}	0.10	1.80×10^{-11}	0.05	8.03×10^{-7}	1.41×10^{-15}	1.59×10^{-36} [4]
	2	rs4274624:C>T	*STAT4*	0.227	-0.32	3.90×10^{-9}	-0.13	6.90×10^{-12}	-0.14	1.63×10^{-35}	4.02×10^{-12}	9.73×10^{-66} [4]
	1	rs17849501:G>T	*NCF2,SMG7*	0.038	0.70	1.05×10^{-11}	0.10	5.00×10^{-2}	0.07	7.93×10^{-4}	1.39×10^{-11}	1.81×10^{-59} [4]
	8	rs998683:G>A	*BLK*	0.260	0.28	1.96×10^{-7}	0.09	1.40×10^{-6}	-0.03	7.29×10^{-3}	9.49×10^{-10}	1.26×10^{-14} [4]
	1	rs6679677:C>A	*RSBN1*	0.140	0.27	3.43×10^{-5}	0.59	3.10×10^{-149}	0.38	1.44×10^{-154}	1.12×10^{-9}	4.54×10^{-13} [4]
	12	rs10774624:G>A	*SH2B3*	0.400	-0.20	4.48×10^{-5}	-0.08	2.40×10^{-7}	-0.21	1.78×10^{-101}	5.39×10^{-9}	1.49×10^{-7} [4]

Supplementary Table 1: The significance results of sfFDR applied to four diseases in the FinnGen biobank. Functional p-values (P_f) and q-values (Q_f) of the lead SNPs when applying sfFDR to autoimmune thyroiditis (ATH; 688 cases and $424{,}208$ controls), juvenile idiopathic arthritis (JIA; 788 cases and $172{,}834$ controls), myositis (MYO; 932 cases and $357{,}549$ controls), and systemic lupus erythematosus (SLE; 835 cases and $232{,}612$ controls) in the FinnGen biobank [5]. The informative traits were rheumatoid arthritis [6] and hypothyroidism [7]. Note that the FinnGen release versions R5 (JIA), R7 (SLE) and R12 (MYO, ATH) were used and the SNP identifiers are given as rsid:reference_allele>effect_allele.

						Informative traits							
				**EGPA**		**ASTAO**		**ASTCO**		**EOSC**			
Chr	rsid	Gene	MAF	β	P	β	P	β	P	β	P	P_f	Q_f
5	rs1837253:C>T	*TSLP*	0.258	−0.41	7.96×10^{-10}	−0.08	1.50×10^{-17}	−0.17	5.50×10^{-37}	−0.04	1.89×10^{-22}	5.35×10^{-14}	4.27×10^{-7}
2	rs144569746:T>C	*BCL2L11*	0.107	−0.57	1.54×10^{-9}	−0.06	1.70×10^{-5}	−0.06	2.80×10^{-3}	−0.06	2.57×10^{-26}	1.51×10^{-12}	6.01×10^{-6}
5	rs10066308:A>G	*IRF1*	0.305	−0.35	6.95×10^{-8}	−0.07	2.30×10^{-13}	−0.09	5.10×10^{-13}	−0.04	8.18×10^{-32}	1.33×10^{-11}	1.18×10^{-5}
10	rs7898135:A>C	*GATA3*	0.283	0.31	2.72×10^{-6}	0.10	1.20×10^{-26}	0.10	1.50×10^{-14}	0.04	6.97×10^{-23}	2.01×10^{-10}	5.74×10^{-5}
6	rs11754356:T>C	*BACH2*	0.394	0.27	7.14×10^{-6}	0.05	2.70×10^{-10}	0.09	1.10×10^{-13}	0.03	4.80×10^{-19}	4.90×10^{-10}	1.12×10^{-4}
21	rs8133843:A>G	*RUNX1*	0.373	−0.30	9.69×10^{-7}	−0.04	4.40×10^{-5}	−0.02	3.90×10^{-2}	−0.03	7.90×10^{-12}	2.95×10^{-9}	3.51×10^{-4}
3	rs9825301:T>G	*TPRG1*	0.314	−0.29	4.05×10^{-6}	−0.03	1.60×10^{-4}	−0.05	1.30×10^{-5}	−0.03	1.51×10^{-14}	6.82×10^{-9}	5.73×10^{-4}
17	rs12952581:A>G	*ZNF652*	0.143	−0.24	7.96×10^{-5}	−0.05	1.90×10^{-7}	−0.09	8.40×10^{-14}	−0.03	2.67×10^{-13}	3.29×10^{-8}	1.39×10^{-3}
12	rs10876864:A>G	*IKZF4*	0.416	0.23	1.19×10^{-4}	0.06	1.40×10^{-12}	0.10	1.10×10^{-17}	0.03	6.24×10^{-13}	3.61×10^{-8}	1.47×10^{-3}
11	rs7927997:T>C	*LRRC32*	0.395	−0.22	2.37×10^{-4}	−0.08	5.20×10^{-19}	−0.17	6.20×10^{-46}	−0.04	7.32×10^{-27}	3.86×10^{-8}	1.53×10^{-3}

Supplementary Table 2: Functional p-values (P_f) and q-values (Q_f) of the lead SNPs from the EGPA analysis. The informative traits were adult-onset asthma (ASTAO), childhood-onset asthma (ASTCO), and eosinophil count (EOSC). The identifiers for the reference SNP cluster ID (rsid) column are given as rsid:reference_allele>effect_allele. The lead SNP chromosome (Chr) position, nearest gene, minor allele frequency (MAF), and p-value (P) are reported. The standard p-values were calculated from a two-sided chi-square test using the mixed model association method BOLT-LMM. We adjusted for multiple comparisons using the Bonferroni correction with a genome-wide significance threshold of 5×10^{-8}.

Gene	sfFDR	Standard
BACH2	66	167
BCL2L11	13	14
GATA3	112	272
IKZF4	45	1,113
IRF1	52	39
LRRC32	136	2,564
RUNX1	141	68
TPRG1	45	64
TSLP	1	1
ZNF652	100	1,093

Supplementary Table 3: Fine-mapping results in the EGPA analysis. The size of the 95% credible set using sfFDR and the standard (or original) p-values in the EGPA study. Note that only *TSLP* and *BCL2L11* are below genome-wide significance level for the standard p-values.

Gene	ASTAO	ASTCO	EOSC
BACH2	0.196	0.205	0.153
BCL2L11	0.968	0.000	0.850
GATA3	0.254	0.058	0.226
IKZF4	0.194	0.343	0.791
IRF1	0.320	0.000	0.000
LRRC32	0.131	0.161	0.112
RUNX1	0.076	0.081	0.000
TPRG1	0.173	0.000	0.822
TSLP	0.000	0.963	0.963
ZNF652	0.567	0.258	0.439

Supplementary Table 4: Comparing the credible sets between the informative studies and EGPA study. The proportion of SNPs in the 95% credible set from sfFDR that overlap with the credible sets from the informative studies (ASTAO, ASTCO, and EOSC).

2 Supplementary figures

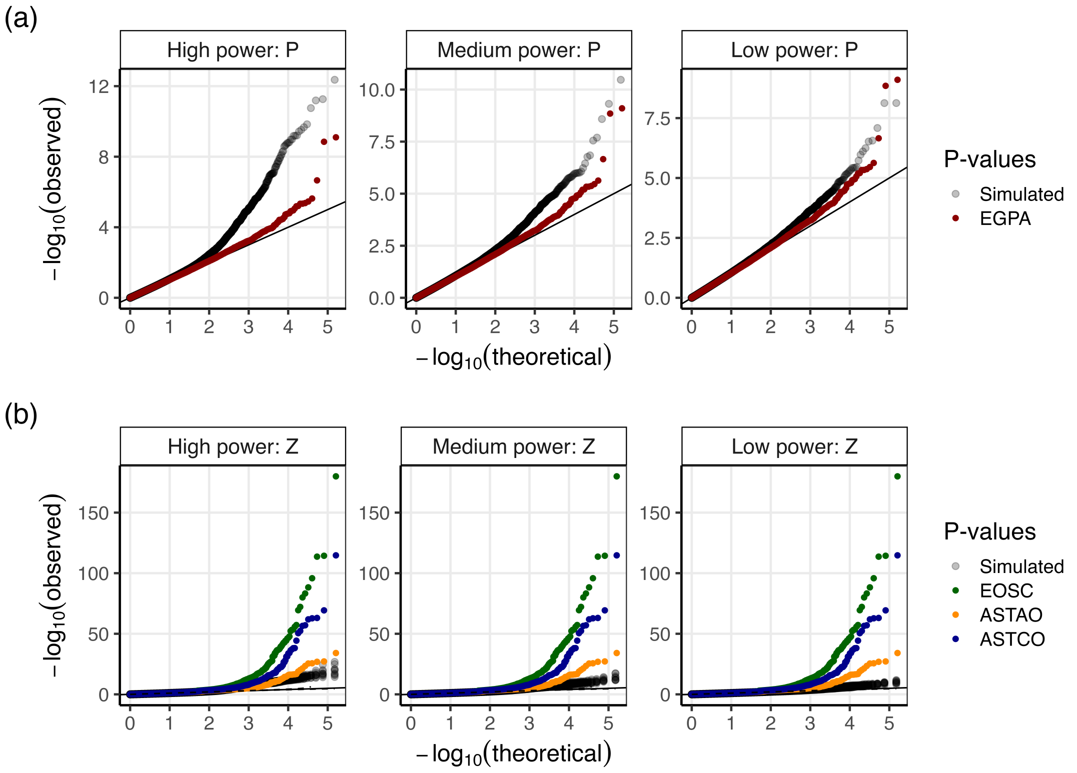

Supplementary Figure 1: A Quantile-Quantile plot of a single realization from the independent SNP simulation study. The power of the (a) primary study and (b) the three informative studies (shown in black) were varied as "High," "Medium," and "Low." We plotted the set of LD-independent SNPs from the EGPA (dark red), eosinophil count (EOSC; dark green), adult-onset asthma (ASTAO; dark orange), and childhood-onset asthma (ASTCO; dark blue) studies for comparison to real data benchmarks.

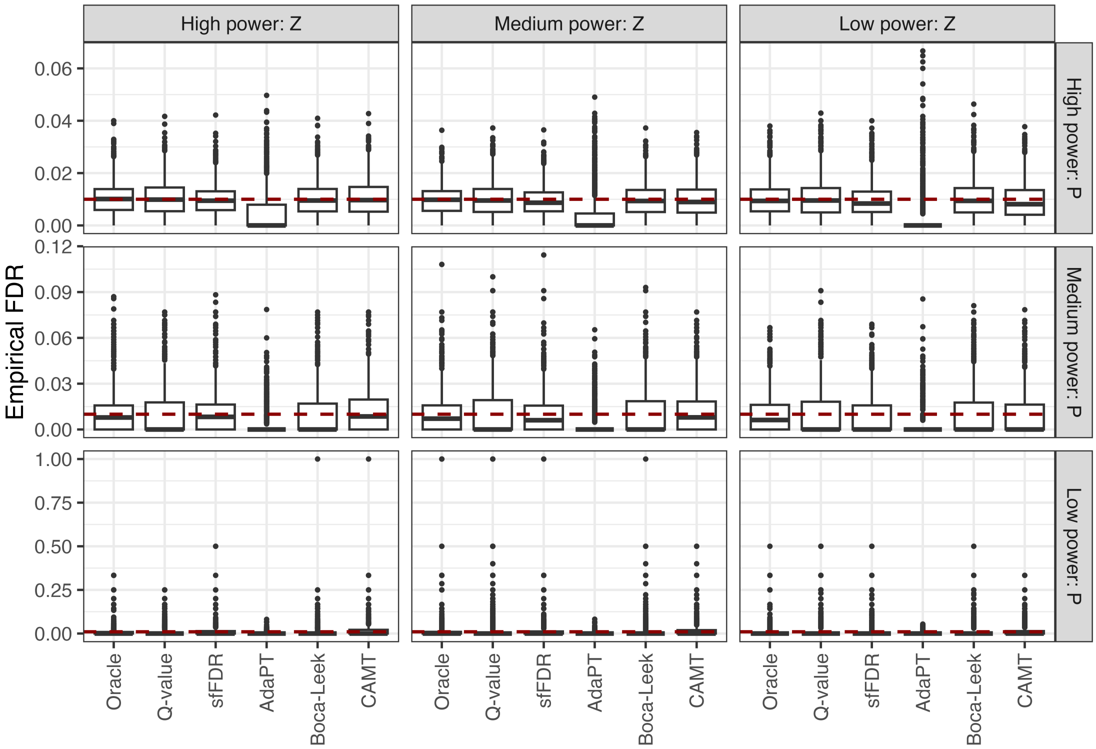

Supplementary Figure 2: Evaluating FDR control in the independent SNP simulation study. Assessing the target FDR at level 0.01 using the oracle functional q-values, standard q-values, functional q-values from sfFDR, Adapt, CAMT, and Boca-Leek. The "None," "Moderate," and "Large" effect size strength settings were combined. The boxplot shows the median (middle black line), first and third quartiles (box limits), $1.5\times$ interquartile range (IQR; whiskers), and outliers (points).

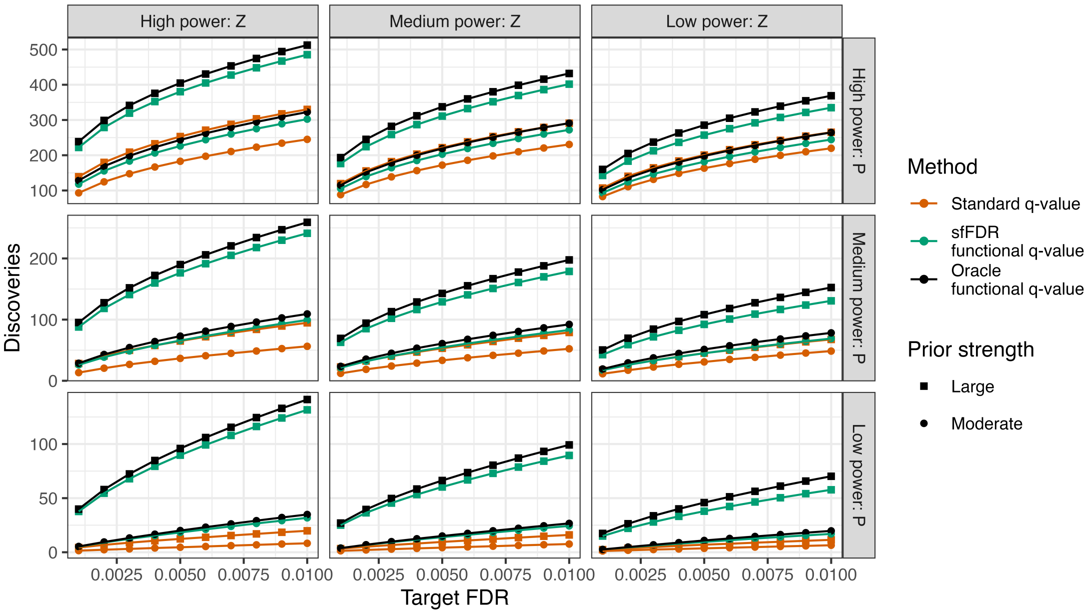

Supplementary Figure 3: Evaluating sfFDR in the independent SNP simulation study. The number of discoveries as a function of the target false discovery rate (FDR) using the standard q-value (dark orange), functional q-value from sfFDR (green), and the oracle functional q-value (black). We varied the power of the primary study (rows), the power of the informative studies (columns), and the effect size strength of the informative studies (shape). Each point is the average from 500 replicates.

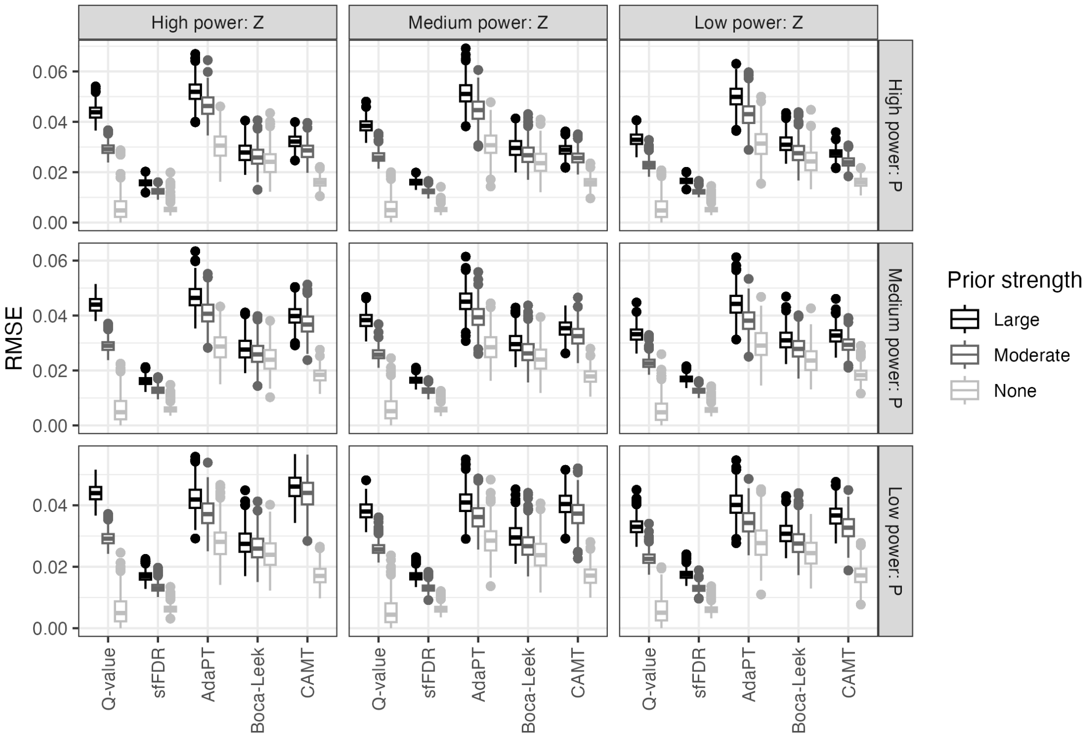

Supplementary Figure 4: Comparing estimators of the functional proportion of truly null tests in the independent SNP simulation study. The root mean square error (RMSE) of the estimated proportion of truly null tests was calculated using the standard q-value, sfFDR, Adapt, CAMT, and Boca-Leek. There were a total of 500 replicates at each combination of primary study power (rows), informative study power (columns), and the effect size strength of the informative studies (color). The boxplot shows the median (middle black line), first and third quartiles (box limits), $1.5\times$ interquartile range (IQR; whiskers), and outliers (points).

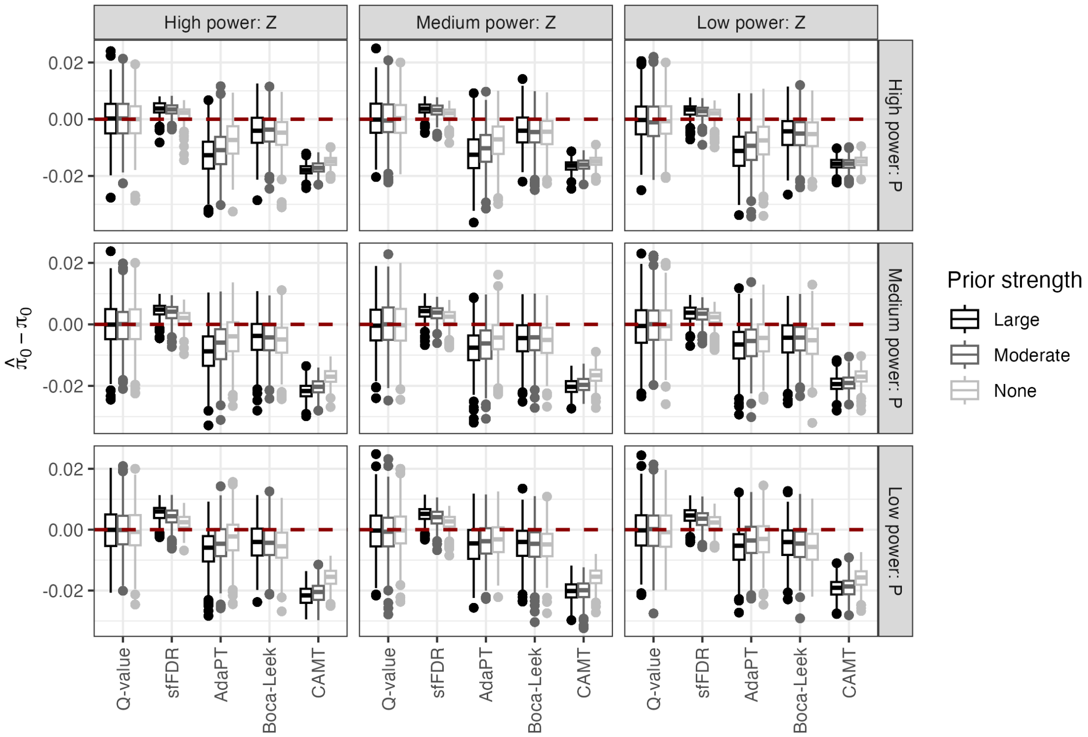

Supplementary Figure 5: Comparing estimators of the proportion of truly null tests in the independent SNP simulation study. The estimated proportion of truly null tests was calculated using the standard q-value, sfFDR, Adapt, CAMT, and Boca-Leek. There were a total of 500 replicates at each combination of primary study power (rows), informative study power (columns), and the effect size strength of the informative studies (color). The boxplot shows the median (middle black line), first and third quartiles (box limits), $1.5\times$ interquartile range (IQR; whiskers), and outliers (points).

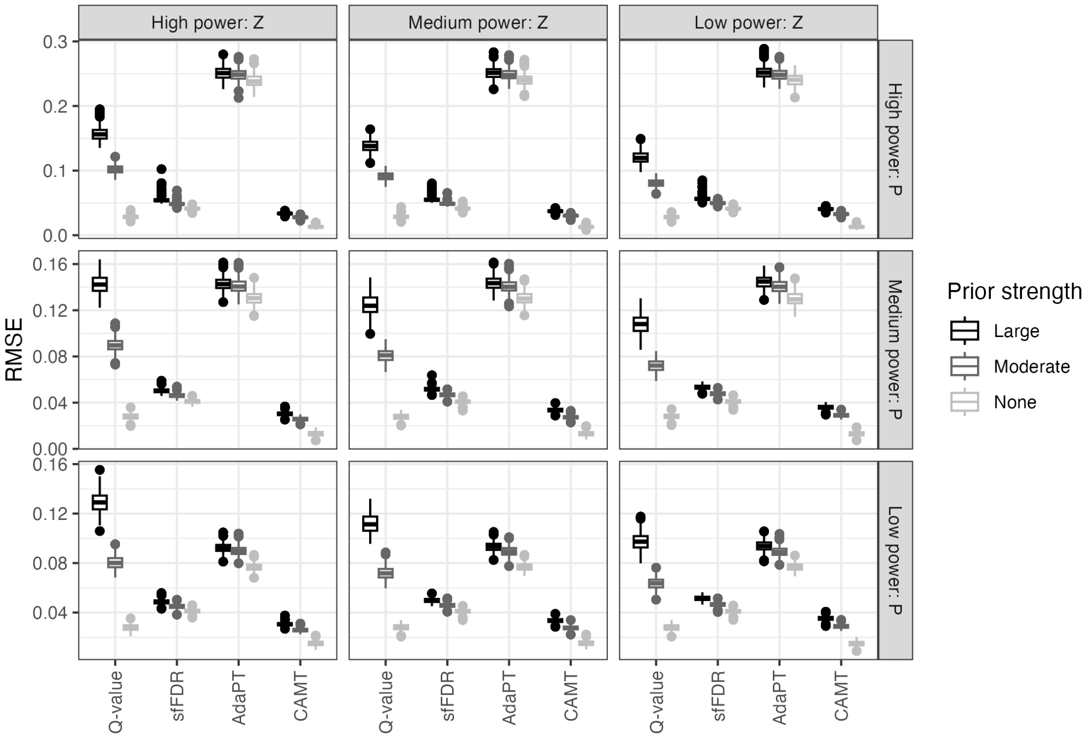

Supplementary Figure 6: Comparing estimators of the conditional density in the independent SNP simulation study. The root mean square error (RMSE) of the log-transformed estimated conditional density of the p-values given the informative traits using the standard q-value (i.e., the marginal density), sfFDR, Adapt, and CAMT. There were a total of 500 replicates at each combination of primary study power (rows), informative study power (columns), and the effect size strength of the informative studies (color). The boxplot shows the median (middle black line), first and third quartiles (box limits), $1.5\times$ interquartile range (IQR; whiskers), and outliers (points).

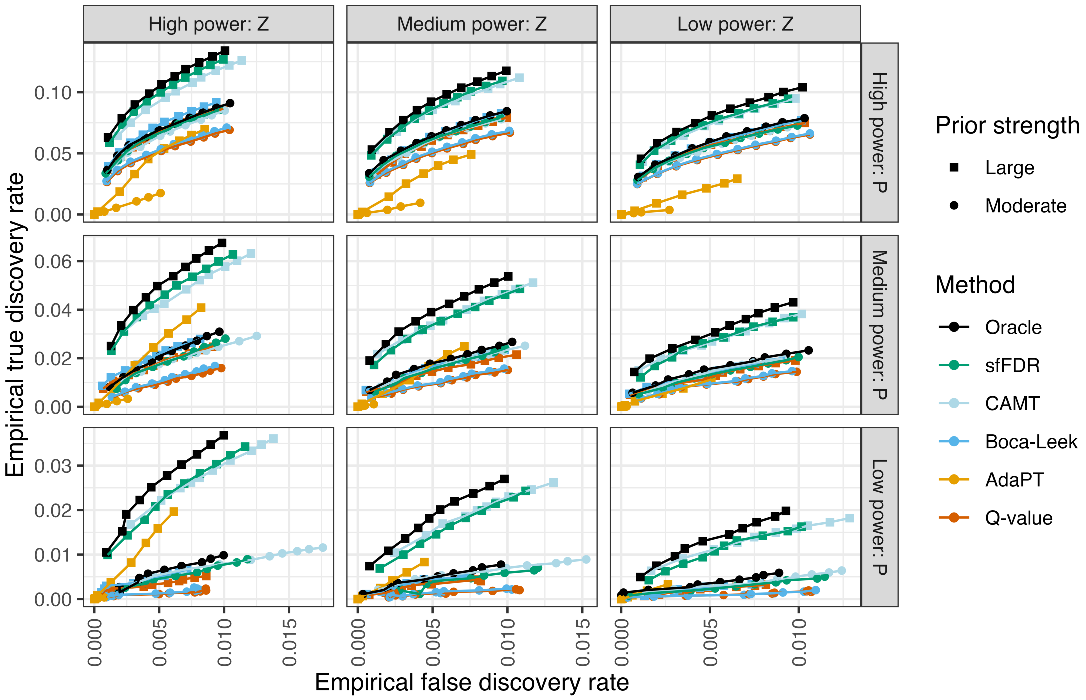

Supplementary Figure 7: Performance of the FDR procedures in the independent SNP simulation study. The empirical true and false discovery rates were calculated at a target FDR level of $0.001, 0.002, \ldots, 0.01$ using the oracle functional q-value (black), functional q-value from sfFDR (green), CAMT (light blue), Boca-Leek (blue), AdaPT (orange), and standard q-value (dark orange). We varied the power of the primary study (rows), the power of the informative studies (columns), and the effect size strength of the informative studies (shape). Each point is the average from 500 replicates.

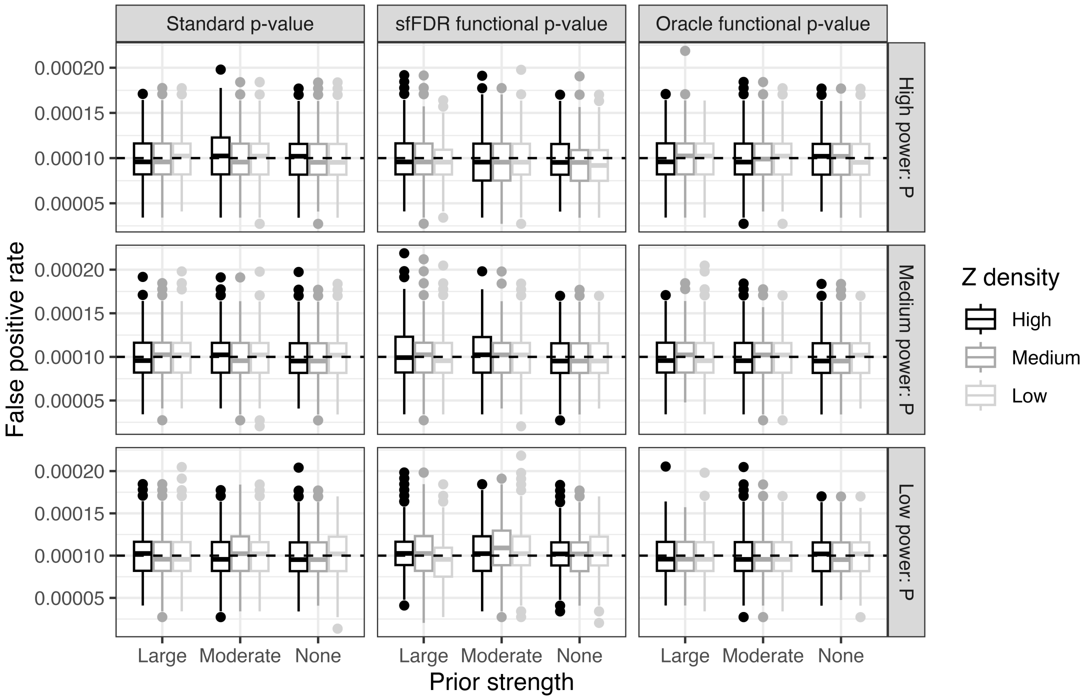

Supplementary Figure 8: Evaluating type I error rate control of the functional p-value in the independent SNP simulation study. The type I error rate was calculated for the standard p-values, functional p-values from sfFDR, and the oracle functional p-values at a significance threshold of 1×10^{-4}. We varied the power of the primary study (rows), the power of the informative studies (color), and the effect size strength of the informative studies (x-axis). There were a total of 500 simulations at each setting. The boxplot shows the median (middle black line), first and third quartiles (box limits), $1.5\times$ interquartile range (IQR; whiskers), and outliers (points).

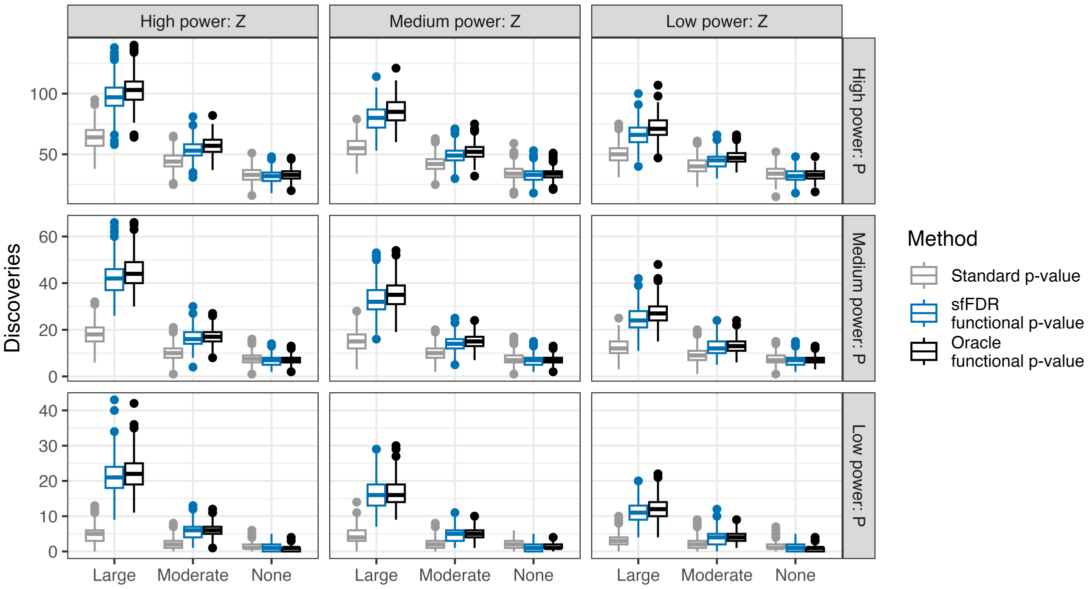

Supplementary Figure 9: Evaluating the functional p-value from sfFDR in the independent SNP simulation study. The number of discoveries was calculated using the standard p-value (grey), functional p-value from sfFDR (blue), and oracle functional p-value (black) at a significance threshold of 5×10^{-8}. We varied the power of the primary study (rows), the power of the informative studies (columns), and the effect size strength of the informative studies (x-axis). There were a total of 500 simulations at each setting. The boxplot shows the median (middle black line), first and third quartiles (box limits), $1.5\times$ interquartile range (IQR; whiskers), and outliers (points).

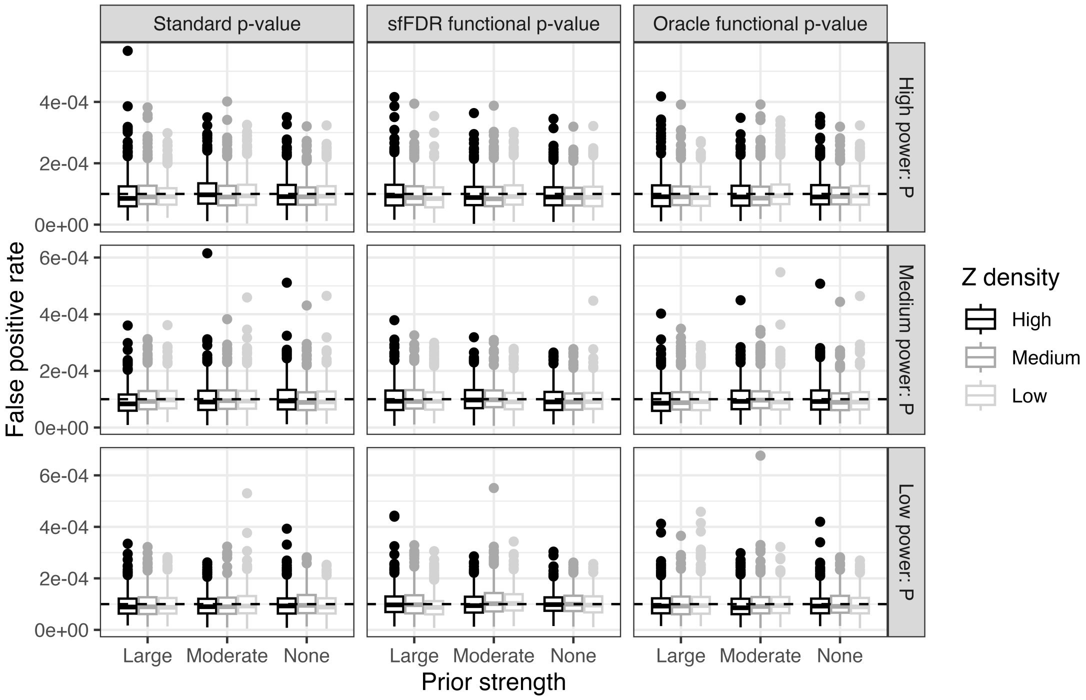

Supplementary Figure 10: Evaluating type I error rate control of the functional p-value in the dependent SNP simulation study. The type I error rate was calculated for the standard p-values, functional p-values, and the oracle functional p-values at a significance threshold of 1×10^{-4}. We varied the power of the primary study (rows), the power of the informative studies (color), and the effect size strength of the informative studies (x-axis). There were a total of 500 simulations at each setting. The boxplot shows the median (middle black line), first and third quartiles (box limits), $1.5\times$ interquartile range (IQR; whiskers), and outliers (points).

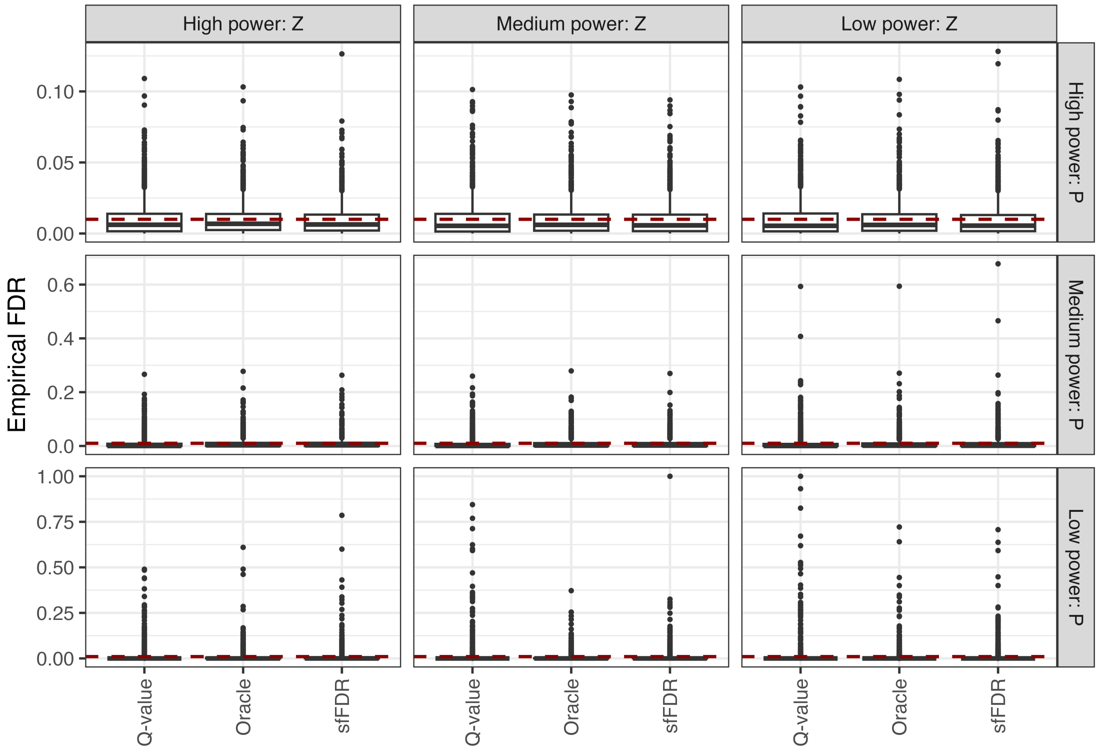

Supplementary Figure 11: Evaluating FDR control in the dependent SNP simulation study. Assessing the target FDR at level 0.01 using the oracle functional q-value, standard q-values, and functional q-values from sfFDR. The "None," "Moderate," and "Large" effect size strength settings were combined. The boxplot shows the median (middle black line), first and third quartiles (box limits), $1.5\times$ interquartile range (IQR; whiskers), and outliers (points).

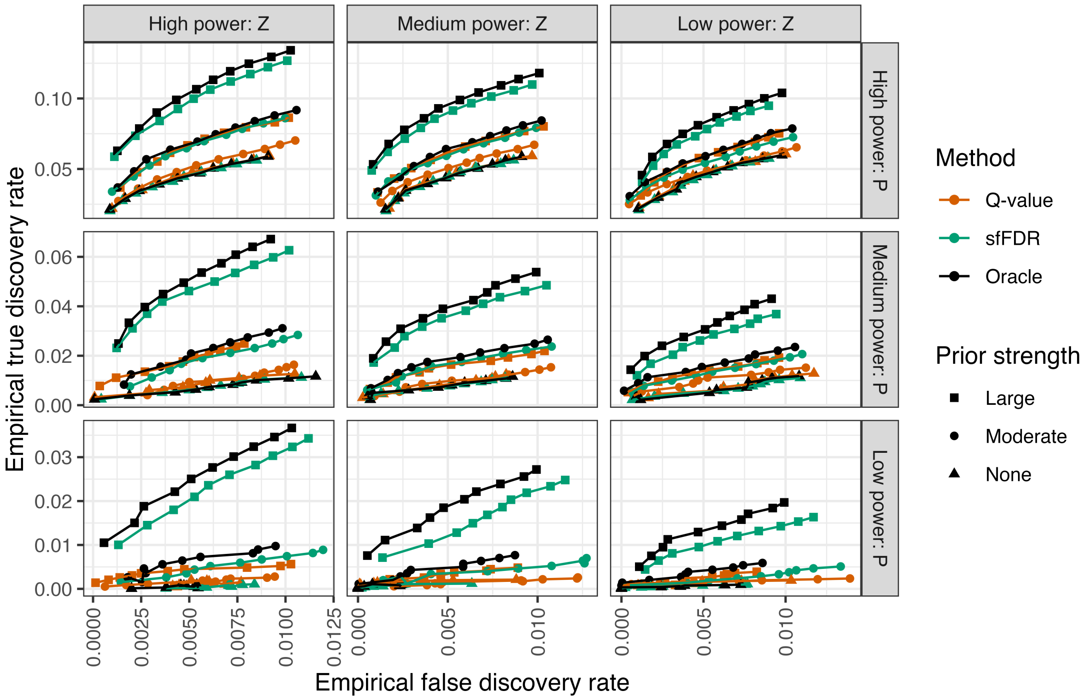

Supplementary Figure 12: Performance of sfFDR in the dependent SNP simulation study. The empirical true and false discovery rates were calculated at a target FDR level of $0.001, 0.002, \ldots, 0.01$ using the standard q-value (dark orange), functional q-value from sfFDR (green), and oracle functional q-value (black). We varied the power of the primary study (rows), the power of the informative studies (columns), and the effect size strength of the informative study (shape). Each point is the average from 500 replicates.

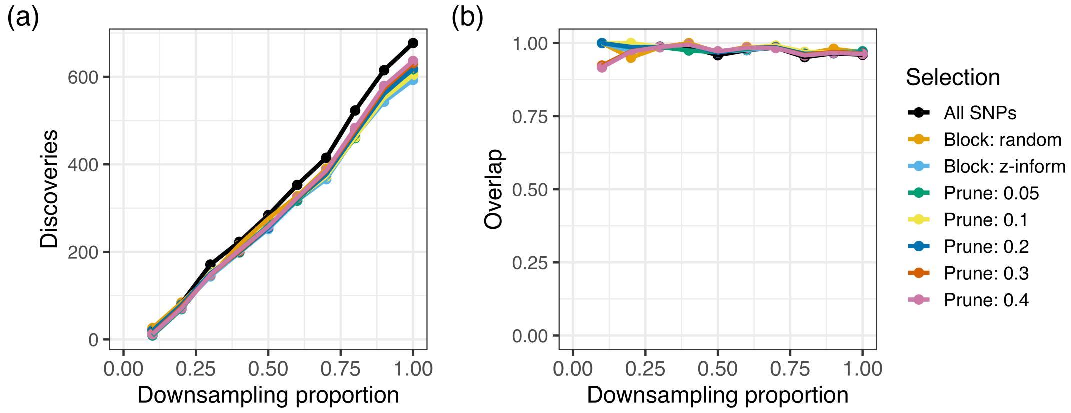

Supplementary Figure 13: Evaluating various SNP selection strategies in sfFDR using the UK Biobank. The informative traits were body fat percentage (BFP), cholesterol, and triglycerides. (a) The number of discoveries as a function of the proportion of the study sample size (i.e., downsampling proportion) at a significance threshold of 5×10^{-8}; (b) the overlap in discoveries (or replication rate) with a meta-analysis approach.

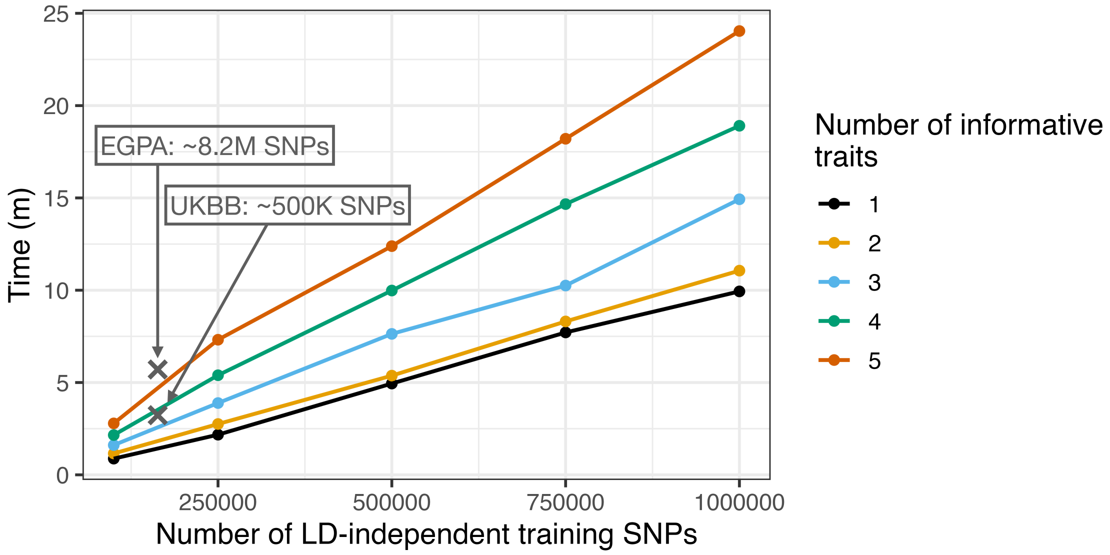

Supplementary Figure 14: The computational time to train sfFDR on a set of LD-independent SNPs as a function of the number of informative traits. The time to train sfFDR on the UKBB study and EGPA study are denoted by 'x,' which also includes the time to predict the left out LD-dependent SNPs. A single core of an Apple M3 processor was used in this analysis.

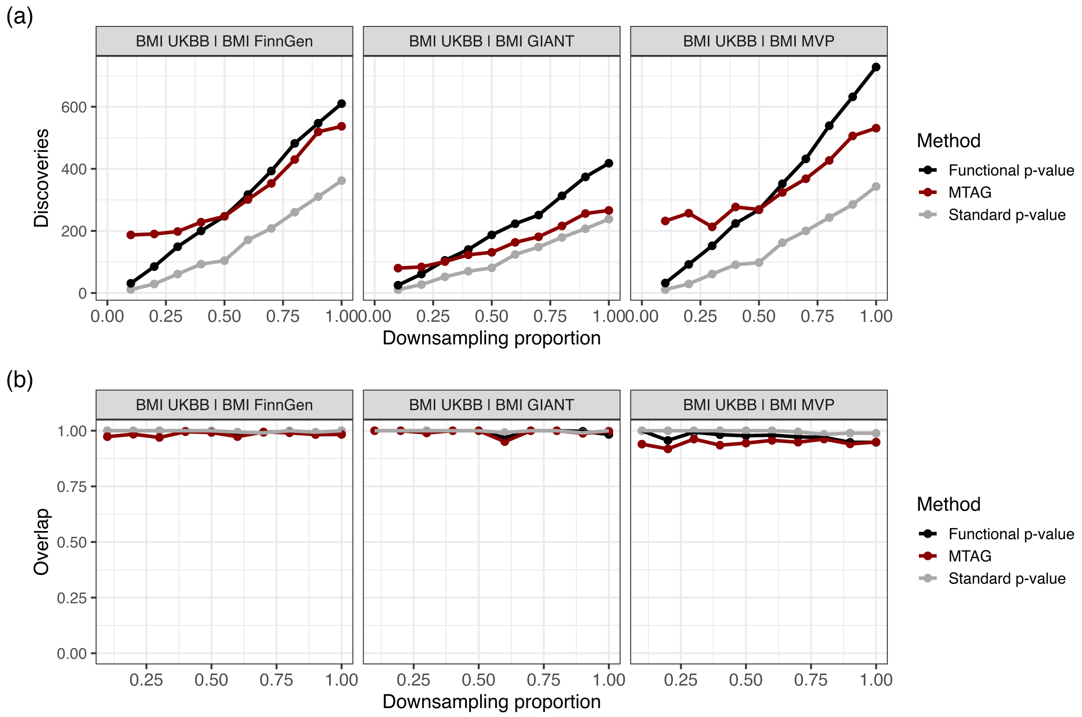

Supplementary Figure 15: Using summary statistics from slightly different populations in sfFDR. We applied sfFDR to the BMI summary statistics in the UK Biobank leveraging BMI summary statistics from either FinnGen (left), GIANT (middle) or Million Veterans Program (MVP, European population; right) biobanks as an informative trait. (a) The number of discoveries as a function of the downsampling proportion. (b) The proportion of discoveries ($p < 5 \times 10^{-8}$) that overlapped with a meta-analysis of the UK Biobank data and either MVP (FinnGen and GIANT) or FinnGen (MVP). sfFDR was trained on 491,999, 461,811, and 166,416 overlapping SNPs with the FinnGen ($500{,}348$ samples), MVP ($424{,}231$ samples), and GIANT ($339{,}224$ samples) biobanks, respectively.

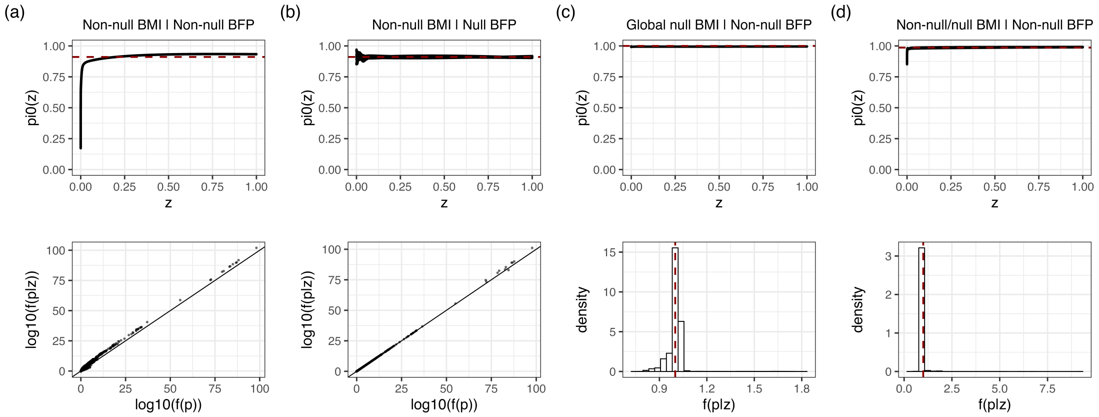

Supplementary Figure 16: The functional proportion of null tests and conditional density estimates from the UK Biobank analysis (downsampling proportion of 1). The functional proportion of null tests (top) and conditional density (bottom) when (a) the primary trait is body mass index (BMI; denoted by p) and the informative trait is body fat percentage (BFP; denoted by z), (b) the primary trait is non-null BMI summary statistics and the informative trait is null BFP summary statistics, (c) the primary trait is null BMI summary statistics and the informative trait is non-null BFP summary statistics, and (d) the primary trait is simulated from a mixture of null and non-null BMI summary statistics and the informative trait is non-null BFP summary statistics. Note that only the null SNPs are shown in (d) and that each point is the average of 10 replicates (except at (a) and the top plot in (b)). The red dashed line denotes either the proportion of truly null tests (top plots) or the expected value (bottom plots).

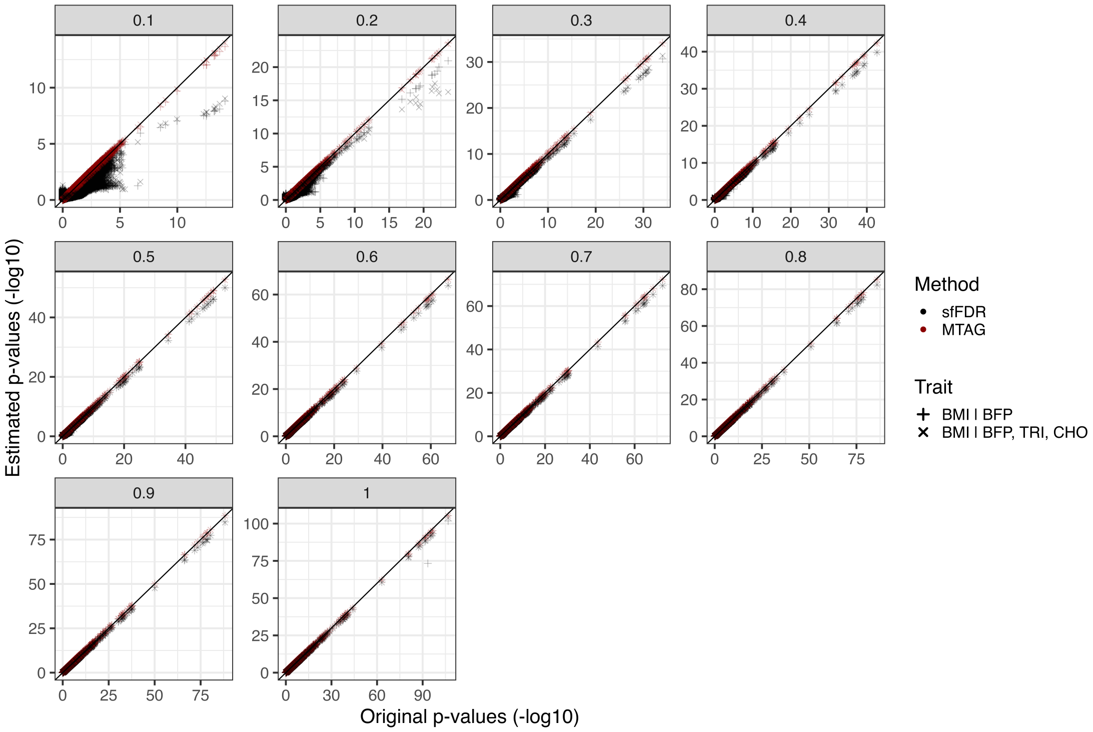

Supplementary Figure 17: Applying sfFDR and MTAG to a null setting where the informative traits were permuted and uncorrelated with BMI. We compared the estimated functional p-values from sfFDR and the estimated p-values from MTAG (color) to the standard p-values (x-axis). There were 10 permutations of the null traits (shape) at each downsampling proportion and each point represents the average functional p-value across permutations. A log10 transformation was applied to both axes.

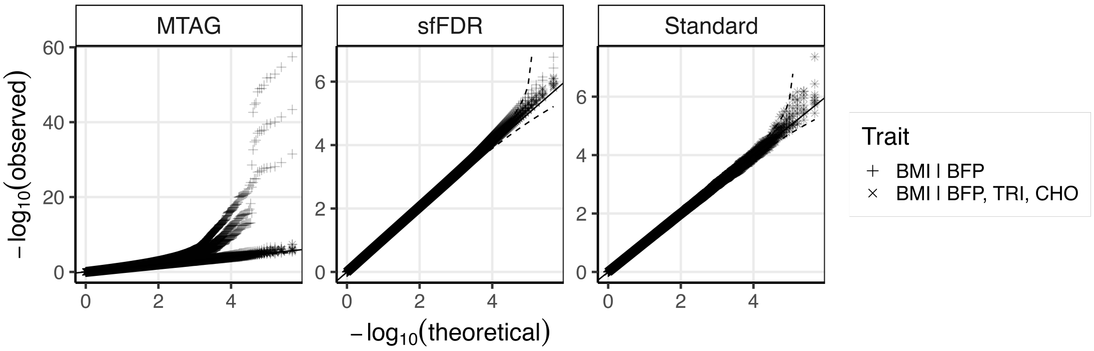

Supplementary Figure 18: Applying sfFDR and MTAG to a null setting where BMI was permuted and uncorrelated with the informative traits. Quantile-Quantile plot of the p-values after applying the standard analysis, sfFDR, and MTAG. There were a total of 10 replicates at each setting (shape).

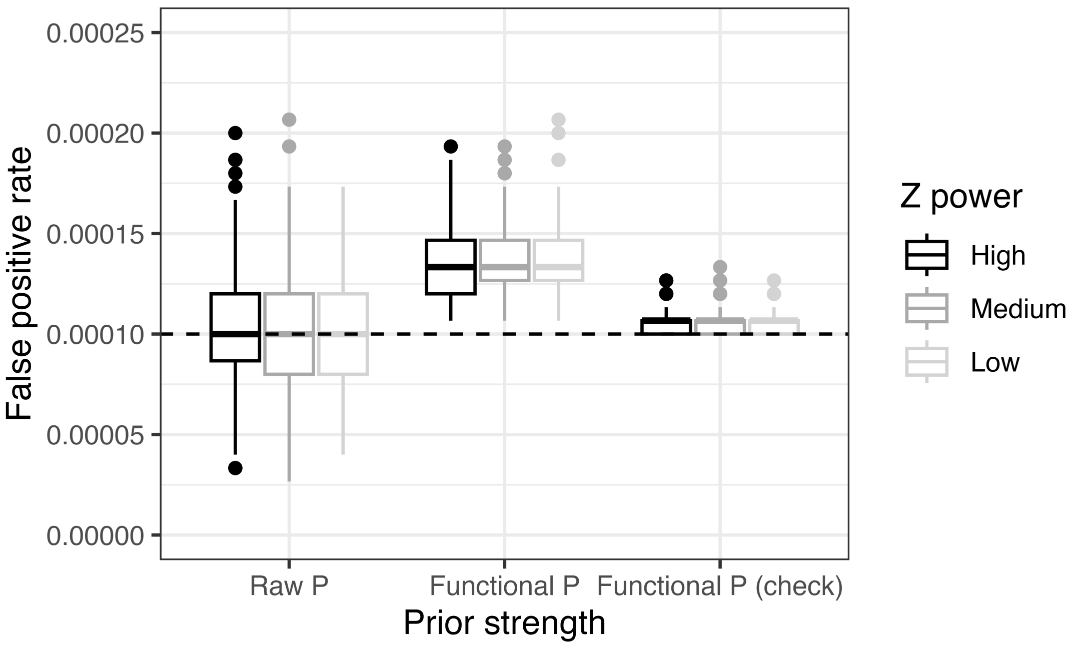

Supplementary Figure 19: Evaluating type I error rate control of the functional p-value under the global null setting. The type I error rate at a significance threshold of 1×10^{-4} using the standard p-values, functional p-values, and functional p-values with $\pi_0(z)$ set to be constant and the nearest neighbor parameter in the density estimator set to be 0.7. We varied the power of the informative studies (color). There were a total of 500 simulations at each setting. The boxplot shows the median (middle black line), first and third quartiles (box limits), $1.5\times$ interquartile range (IQR; whiskers), and outliers (points).

(a)

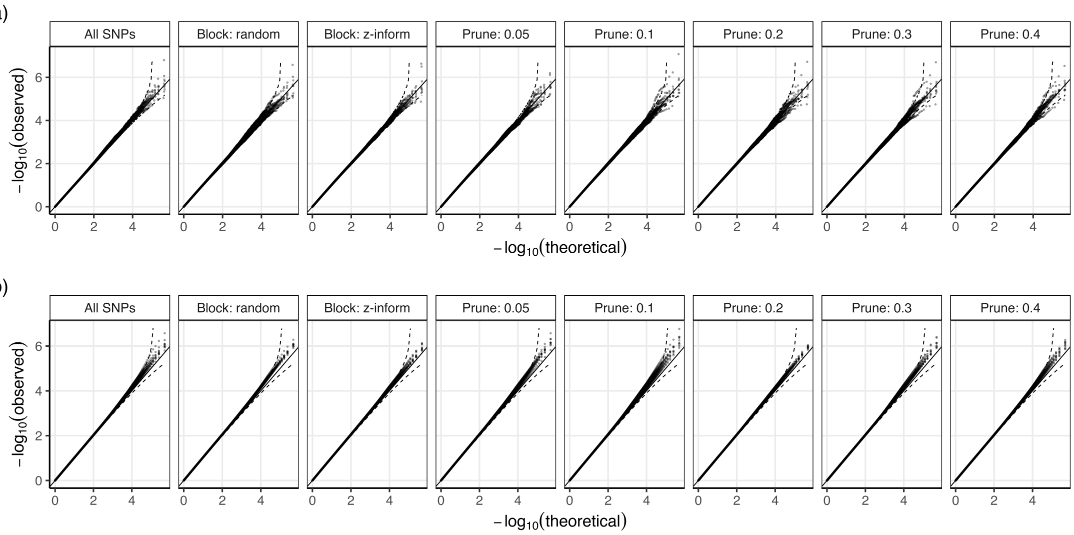

(b)

Supplementary Figure 20: Quantile-Quantile plot of the functional p-values from various SNP selection procedures to train sfFDR in the UK Biobank study. (a) A set of known simulated null SNPs added to 19 random chromosomes in the primary trait (BMI) and not the informative traits (BFP, cholesterol, and triglycerides). (b) The primary trait (BMI) was permuted to be null and uncorrelated with the informative traits (BFP, cholesterol, and triglycerides), i.e., $\pi_0 = 1$. There were 10 replicates in each setting.

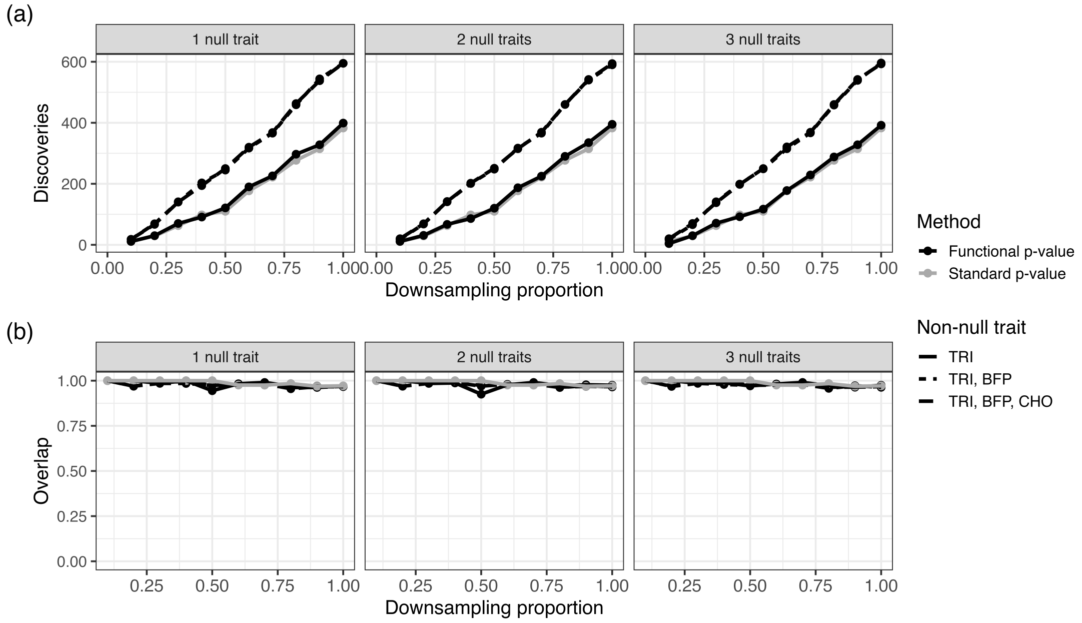

Supplementary Figure 21: Evaluating sfFDR when the informative traits are a mixture of null and non-null in the UK Biobank study. We applied sfFDR to BMI using the informative traits body fat percentage (BFP), cholesterol (CHO), triglycerides (TRI), and a number of null (or uninformative) traits. The traits were fitted in sequence (linetype) where a set of uninformative traits (columns) were added to assess the performance under a mixture of informative and uninformative traits. (a) The number of discoveries as a function of the downsampling proportion. (b) The overlap in discoveries (or replication rate) with a meta-analysis approach.

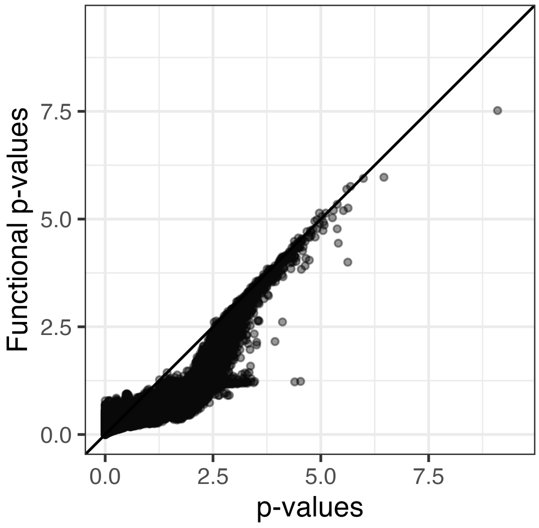

Supplementary Figure 22: Comparison of the functional p-values from sfFDR to the standard p-values in the EGPA study when the informative traits are the UK Biobank null traits. Each point is the average of 10 replicates. A log10 transformation was applied to both axes.

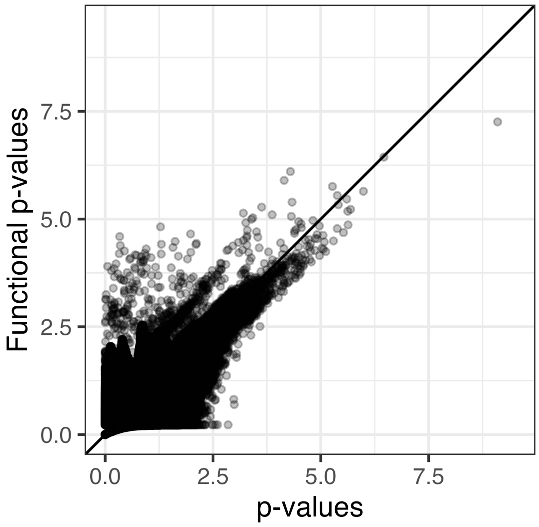

Supplementary Figure 23: Comparison of the functional p-values from sfFDR to the standard p-values in the EGPA study when the informative traits are the UK Biobank obesity-related traits. A log10 transformation was applied to both axes.

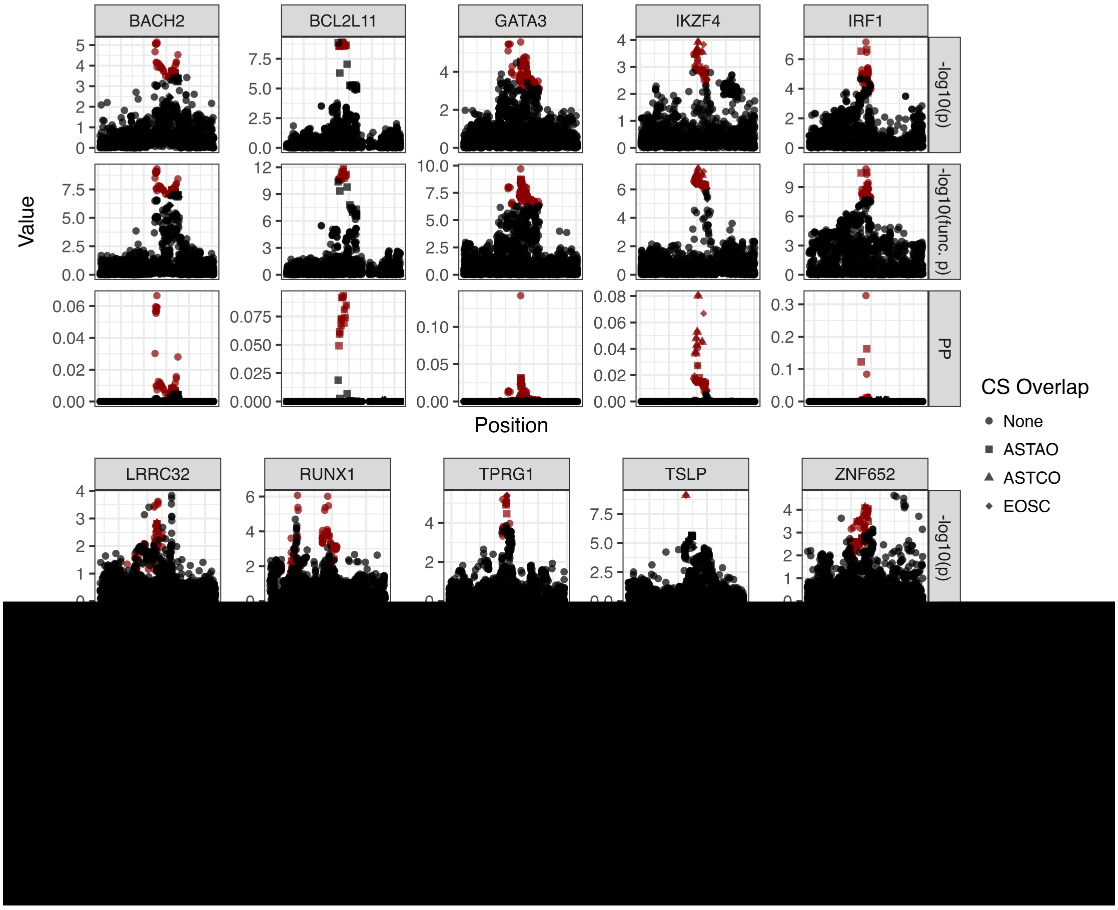

Supplementary Figure 24: Fine-mapping results using the functional local FDR estimates from sfFDR. For each lead SNP, the 95% credible set (CS) is shown in red for EGPA including SNPs 500kb upstream and downstream of the lead SNPs. The top plot in each set shows the local Manhattan plot while the bottom plot shows the fine mapping posterior probabilities calculated under the assumption of a single causal variant. We distinguish the SNPs in the CS that also overlap with the CS from ASTAO (square), ASTCO (triangle), and EOSC (diamond).

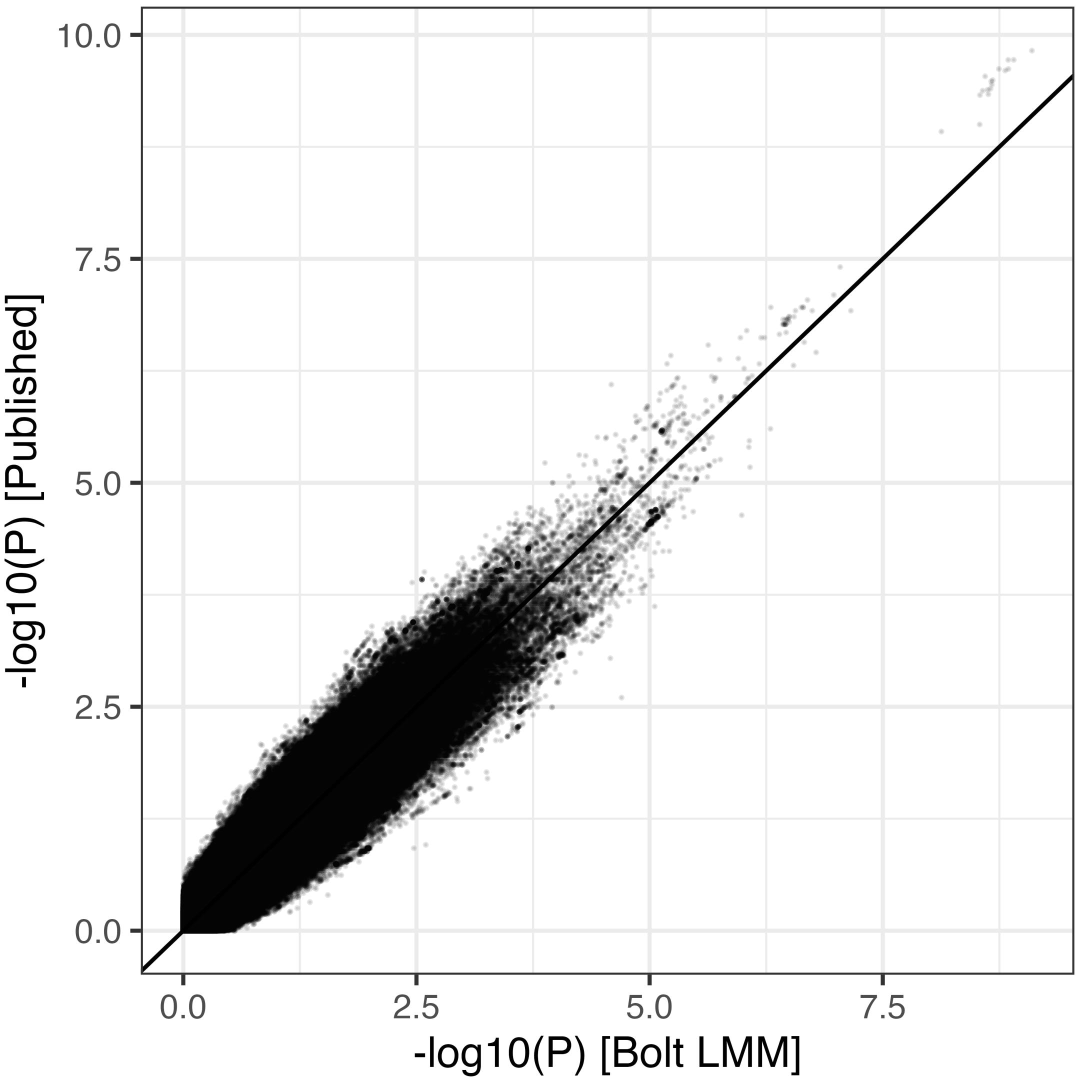

Supplementary Figure 25: Comparison of the published p-values from the EGPA study and the p-values used in sfFDR. Quantile-Quantile plot of the published (discrete) p-values (y-axis) and the p-values used in sfFDR which were calculated using a linear mixed model (y-axis).

References

1. Sakaue S, Kanai M, Tanigawa Y, Karjalainen J, Kurki M, Koshiba S, et al. A cross-population atlas of genetic associations for 220 human phenotypes. Nature Genetics. 2021;53(10):1415–1424. doi:10.1038/s41588-021-00931-x.

2. López-Isac E, Smith SL, Marion MC, Wood A, Sudman M, Yarwood A, et al. Combined genetic analysis of juvenile idiopathic arthritis clinical subtypes identifies novel risk loci, target genes and key regulatory mechanisms. Annals of the Rheumatic Diseases. 2021;80(3):321–328. doi:10.1136/annrheumdis-2020-218481.

3. Rothwell S, Amos CI, Miller FW, Rider LG, Lundberg IE, Gregersen PK, et al. Identification of Novel Associations and Localization of Signals in Idiopathic Inflammatory Myopathies Using Genome-Wide Imputation. Arthritis & Rheumatology. 2023;75(6):1021–1027. doi:https://doi.org/10.1002/art.42434.

4. Bentham J, Morris DL, Cunninghame Graham DS, Pinder CL, Tombleson P, Behrens TW, et al. Genetic association analyses implicate aberrant regulation of innate and adaptive immunity genes in the pathogenesis of systemic lupus erythematosus. Nature Genetics. 2015;47(12):1457–1464. doi:10.1038/ng.3434.

5. Kurki MI, Karjalainen J, Palta P, Sipilä TP, Kristiansson K, Donner KM, et al. FinnGen provides genetic insights from a well-phenotyped isolated population. Nature. 2023;613(7944):508–518. doi:10.1038/s41586-022-05473-8.

6. Okada Y, Wu D, Trynka G, Raj T, Terao C, Ikari K, et al. Genetics of rheumatoid arthritis contributes to biology and drug discovery. Nature. 2014;506(7488):376–381. doi:10.1038/nature12873.

7. Mbatchou J, Barnard L, Backman J, Marcketta A, Kosmicki JA, Ziyatdinov A, et al. Computationally efficient whole-genome regression for quantitative and binary traits. Nature Genetics. 2021;53(7):1097–1103. doi:10.1038/s41588-021-00870-7.
