# Supplementary material for: Exploiting pleiotropy to enhance variant discovery with functional false discovery rates
Source: Nat Comput Sci. 2025 Aug 22;5(9):769–81. doi: 10.1038/s43588-025-00852-3 (PMC12457191; doi:10.1038/s43588-025-00852-3)
Supplement: Supplementary file 2 — Reporting Summary [file 43588_2025_852_MOESM2_ESM.pdf]

nature portfolio

Corresponding author(s): Andrew J. Bass
Chris Wallace

Last updated by author(s): Jun 11, 2025

Reporting Summary

Nature Portfolio wishes to improve the reproducibility of the work that we publish. This form provides structure for consistency and transparency in reporting. For further information on Nature Portfolio policies, see our Editorial Policies and the Editorial Policy Checklist.

Statistics

For all statistical analyses, confirm that the following items are present in the figure legend, table legend, main text, or Methods section.

n/a	Confirmed	
☐	☒	The exact sample size (*n*) for each experimental group/condition, given as a discrete number and unit of measurement
☐	☒	A statement on whether measurements were taken from distinct samples or whether the same sample was measured repeatedly
☐	☒	The statistical test(s) used AND whether they are one- or two-sided<br>*Only common tests should be described solely by name; describe more complex techniques in the Methods section.*
☐	☒	A description of all covariates tested
☐	☒	A description of any assumptions or corrections, such as tests of normality and adjustment for multiple comparisons
☐	☒	A full description of the statistical parameters including central tendency (e.g. means) or other basic estimates (e.g. regression coefficient) AND variation (e.g. standard deviation) or associated estimates of uncertainty (e.g. confidence intervals)
☐	☒	For null hypothesis testing, the test statistic (e.g. *F*, *t*, *r*) with confidence intervals, effect sizes, degrees of freedom and *P* value noted<br>*Give P values as exact values whenever suitable.*
☒	☐	For Bayesian analysis, information on the choice of priors and Markov chain Monte Carlo settings
☒	☐	For hierarchical and complex designs, identification of the appropriate level for tests and full reporting of outcomes
☒	☐	Estimates of effect sizes (e.g. Cohen's *d*, Pearson's *r*), indicating how they were calculated

Our web collection on statistics for biologists contains articles on many of the points above.

Software and code

Policy information about availability of computer code

Data collection	There was no software used in data collection.
Data analysis	We used the publicly available software plink (v2.0; https://www.cog-genomics.org/plink/2.0/) and R (v4.4.3). We used the R packages sffdr (v1.0.0; https://github.com/ajbass/sffdr), CAMT (v1.1; https://github.com/jchen1981/CAMT), adaptMT (v1.0.0), swfdr (v1.34.0), qvalue (v2.38.0), tidyverse (v2.0.0), patchwork (v1.3.0), and coloc (v5.2.3). The code to reproduce the results in the manuscript is available at https://github.com/ajbass/sffdr_manuscript.

For manuscripts utilizing custom algorithms or software that are central to the research but not yet described in published literature, software must be made available to editors and reviewers. We strongly encourage code deposition in a community repository (e.g. GitHub). See the Nature Portfolio guidelines for submitting code & software for further information.

Data

Policy information about availability of data

All manuscripts must include a data availability statement. This statement should provide the following information, where applicable:

- Accession codes, unique identifiers, or web links for publicly available datasets
- A description of any restrictions on data availability
- For clinical datasets or third party data, please ensure that the statement adheres to our policy

The asthma (GCST007800, GCST007799), eosinophil count (GCST004606), rheumatoid arthritis (GCST002318), hypothyroidism (GCST90013893) and EGPA

(GCST009250) GWAS summary statistics are publicly available to download at https://www.ebi.ac.uk/gwas. The MYO, SLE, JIA, and ATH GWAS summary statistics are publicly available to download at https://finngen.gitbook.io/data-download.
The Million Veteran Program summary statistics can be downloaded through dbGAP (accession number phs002453).
Access to the UK Biobank data can be requested at https://www.ukbiobank.ac.uk/enable-your-research/apply-for-access.

Human research participants

Policy information about studies involving human research participants and Sex and Gender in Research.

Reporting on sex and gender	All analyses included males and females. We report that sex was included as a covariate in association analyses.
Population characteristics	The average age of the UK Biobank is 57 where 54% of participants are female. The average age of the MVP Biobank is 67 where 8% are female. The median age of FinnGen Biobank is 53 where 57% are female.
Recruitment	The UK Biobank recruited half a million volunteers 40–69 years old in the United Kingdom across 22 assessment centers from 2006 to 2010. See https://www.ukbiobank.ac.uk/media/gnkeyh2q/study-rationale.pdf for additional details. The FinnGen Biobank recruited over 500,000 individuals (release 12) and the MVP Biobank enrolled over 450,000 (version 11) individuals. See https://www.finngen.fi/en/node/1985 and https://www.ncbi.nlm.nih.gov/projects/gap/cgi-bin/study.cgi?study_id=phs001672.v11.p1 for recruitment details for FinnGen and MVP Biobanks, respectively.
Ethics oversight	The research in this work abides by the Helsinki Declaration. The North West Multi-centre Research Ethics Committee (MREC) approved the protocols of the UK Biobank study. The REC reference number is 06/MRE08/65. The FinnGen Biobank collected informed consent for research via the Finnish Biobank Act. The Central VA Institutional Review Board (IRB) provided appropriate consent and protocol approvals for the MVP Biobank.

Note that full information on the approval of the study protocol must also be provided in the manuscript.

Field-specific reporting

Please select the one below that is the best fit for your research. If you are not sure, read the appropriate sections before making your selection.

☒ Life sciences ☐ Behavioural & social sciences ☐ Ecological, evolutionary & environmental sciences

For a reference copy of the document with all sections, see nature.com/documents/nr-reporting-summary-flat.pdf

Life sciences study design

All studies must disclose on these points even when the disclosure is negative.

Sample size	No sample size calculation was performed. We analyzed available participants in the UK Biobank (sample size: 380,600). The summary statistics for the MVP, FinnGen, asthma, eosinophil count, EGPA, rheumatoid arthritis, and hypothyroidism studies were previously published.
Data exclusions	In the UK Biobank study, there were a few individuals that withdrew consent. We removed individuals of non-British descent and any individuals that were related. This information was provided by the UK Biobank. We also removed individuals with a sex chromosome aneuploidy, and genotype missing rate >0.05. After filtering, there were 380,600 individuals in our analysis.
Replication	We searched previous GWAS findings of immune-related traits to determine whether significant loci were previously known. There was no experimental replication attempted.
Randomization	The data used was from observation studies and so randomization was not applicable to our study. We regressed out covariates such as sex, age, and the top 20 PCs to account for ancestry in the UK Biobank study. The other studies were also observational studies where ancestry and other observed covariates were accounted for in the testing procedure.
Blinding	Blinding was not applicable in our study as we used coded de-identified data.

Reporting for specific materials, systems and methods

We require information from authors about some types of materials, experimental systems and methods used in many studies. Here, indicate whether each material, system or method listed is relevant to your study. If you are not sure if a list item applies to your research, read the appropriate section before selecting a response.

Materials & experimental systems

n/a	Involved in the study
☒	☐ Antibodies
☒	☐ Eukaryotic cell lines
☒	☐ Palaeontology and archaeology
☒	☐ Animals and other organisms
☒	☐ Clinical data
☒	☐ Dual use research of concern

Methods

n/a	Involved in the study
☒	☐ ChIP-seq
☒	☐ Flow cytometry
☒	☐ MRI-based neuroimaging
